# Quantitative Analysis of Pseudogene-Associated Errors During Germline Variant Calling

**DOI:** 10.3390/ijms26010363

**Published:** 2025-01-03

**Authors:** Artem Podvalnyi, Arina Kopernik, Mariia Sayganova, Mary Woroncow, Gauhar Zobkova, Anna Smirnova, Anton Esibov, Andrey Deviatkin, Pavel Volchkov, Eugene Albert

**Affiliations:** 1 Federal Research Center for Innovator and Emerging Biomedical and Pharmaceutical Technologies, 125315 Moscow, Russiaandreideviatkin@gmail.com (A.D.); 2 Faculty of Computer Science, HSE University, 101000 Moscow, Russia; 3 Faculty of Fundamental Medicine, Lomonosov Moscow State University, 119991 Moscow, Russia; 4 Evogen LLC, 115191 Moscow, Russia

**Keywords:** processed pseudogenes, SNPs, ACMG

## Abstract

A pseudogene is a non-functional copy of a protein-coding gene. Processed pseudogenes, which are created by the reverse transcription of mRNA and subsequent integration of the resulting cDNA into the genome, being a major pseudogene class, represent a significant challenge in genome analysis due to their high sequence similarity to the parent genes and their frequent absence in the reference genome. This homology can lead to errors in variant identification, as sequences derived from processed pseudogenes can be incorrectly assigned to parental genes, complicating correct variant calling. In this study, we quantified the occurrence of variant calling errors associated with pseudogenes, generated by the most popular germline variant callers, namely GATK-HC, DRAGEN, and DeepVariant, when analysing 30x human whole-genome sequencing data (n = 13,307). The results show that the presence of pseudogenes can interfere with variant calling, leading to false positive identifications of potentially clinically relevant variants. Compared to other approaches, DeepVariant was the most effective in correcting these errors.

## 1. Introduction

Pseudogenes are genomic DNA sequences that resemble normal genes but are non-functional; they are considered evolutionary remnants of genes that were once active [1]. Among them, processed pseudogenes (now PPs) are particularly numerous. They are formed by the reverse transcription of mRNA transcripts, followed by integration of the resulting cDNA into the genome. PPs lack introns and regulatory regions but show high sequence similarity to their parental genes [2]. This high homology represents a major challenge in genome analyses, especially in the calling of genetic variants [3]. Many pseudogenes are absent from the reference genome, making the correct mapping of short reads virtually impossible. A recent study [4] estimated that 2/3rds of individuals harbour at least one non-reference PP. Moreover, the distribution of novel pseudogenes is population-specific [5], therefore requiring wide ethnic studies for correct description.

The precise determination of germline variants is crucial for the identification of clinically relevant genetic alterations that can have an impact on the diagnosis and prognosis of diseases, as well as on therapeutic strategies. High-throughput sequencing (HTS) technologies have revolutionised this field, enabling the detection of single nucleotide polymorphisms (SNPs) and small insertions or deletions (indels) throughout the genome. The rapid increase in the volume of genomic data poses the question of standardisation and automatization of result interpretation in terms of clinical significance. The most widely used guidelines for variant interpretation in terms of clinical significance are the American College of Medical Genetics and Genomics (ACMG) criteria, which were initially published in 2015 [6]. These guidelines allow for the classification of genetic variants into five categories from pathogenic (P) to likely pathogenic (LPV), variants of uncertain significance (VUS), likely benign, and benign using a set of well-defined criteria. Several tools were designed to automate ACMG-based classification and streamline the processing of genetic data, reducing manual labour [7,8,9].

An increased volume of sequencing and automatization of result interpretation requires a higher quality of analysis to minimise manual corrections. However, the presence of PPs can lead to the misalignment of sequencing reads, as far as reads originating from PPs can be incorrectly assigned to their parental genes, leading to false positive variant calls and making the detection of true pathogenic variants more difficult.

Previous studies have highlighted the impact of genomic duplications and repetitive sequences on variant calling accuracy [10], but a comprehensive analysis focusing on pseudogene-associated errors in different variant calling pipelines is lacking. Understanding the extent to which PPs affect variant calling can support the development of strategies to overcome these errors.

In this study, we quantitatively analysed the occurrence of pseudogene-associated errors predicted by three widely used germline variant calling pipelines, GATK HaplotypeCaller [11], DRAGEN [12], and DeepVariant [13], when processing 30× human whole-genome sequencing data. The results show that PPs affect the accuracy of variant calling and lead to false positive identifications of clinically relevant variants, providing insight into the detection of these errors.

## 2. Results

### 2.1. Identification of Processed Pseudogenes in 13,307 WGS Samples

**Figure 1:**
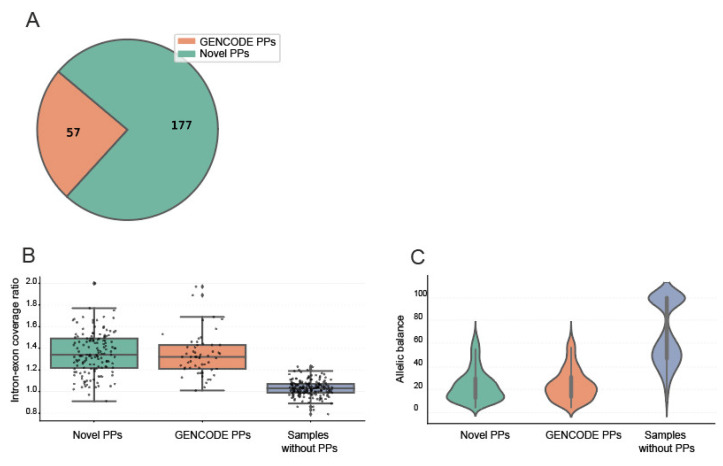
Validation of the identified PPs. (**A**) Number of PPs present in GENCODE among all detected PPs. (**B**). Boxplots of the average exon–intron coverage ratio in samples with PPs for genes with known and novel PPs and in control samples without PPs. Each point on the chart represents the average ratio for all samples for a particular gene within the respective subgroup. (**C**). The violin plots show the allelic balance for genetic variants located in genes associated with PPs and random genetic variants from samples without PPs. The left and middle plots show the allelic balance in the samples with genes with known and novel PPs and the right plot shows the allelic balance for 500 random variants from samples without the PPs.

The first step of the study was to identify PPs within the analysed cohort (Figure S1). To do this, we took advantage of the fact that structural variant detection tools, such as Smoove, can indicate the presence of PPs based on their influence on the mapping of reads. The presence of PPs leads to the presence of split and mismatched reads in the alignment, covering only exonic regions (Figure S2). This feature leads to structural variation detection programmes identifying intronic deletions in genes that contain corresponding PPs. Therefore, we screened all genes for the presence of intronic deletions and labelled those likely to contain a processed pseudogene. This approach was systematically applied to all 13,307 VCF (Variant Call Format) files generated by Smoove. The analysis resulted in the creation of a comprehensive database cataloguing genes with potential PPs in all samples (Table S1). A substantial proportion of the identified PPs (57 out of 234) were already annotated by the GENCODE project (v46) (Figure 1A, Table S1). However, the majority of genes identified by the algorithm (177 of 234) had not been previously annotated with PPs in the reference genome.

To support the newly discovered PPs, we analysed their coverage pattern and compared it with the coverage pattern of genes with PPs listed in GENCODE. We calculated the intron–exon coverage ratio for both groups of genes in samples with and without PPs (Figure 1B). The mean ratio in samples with PPs in newly discovered and in listed GENCODE groups was 1.35 and 1.33, respectively, while the mean ratio for samples without PPs was significantly lower at −1.03, indicating the presence of additional reads aligned to the parent gene exons originating from the PP.

In addition, we analysed the allelic balance (AB) of the genetic variants in the same groups of genes as for the intron–exon coverage ratio for samples with and without PPs (Figure 1C). AB refers to the proportion of alternative reads relative to the total reads at a given genetic locus. In groups of samples with PPs, we found that the mean AB was 24.3% and 23.5% for GENCODE-listed PPs and newly discovered PPs, respectively. In contrast, for the set of random variants in samples without the presence of PPs, the AB followed a bimodal distribution, reflecting the presence of homo- and heterozygous variants with peak values of 100% and 50%, respectively. The observed AB pattern demonstrates that samples with PPs harbour on average 5-times-more variants in the corresponding gene and that these excessive variants are coming from the PPs.

In total, we successfully identified PPs in structural variant data from 13,307 whole-genome sequencing (WGS) samples analysed with Smoove.

### 2.2. Processed Pseudogenes Harbour Deleterious Variants Falsely Assigned to Parent Genes

To confirm our hypothesis that the presence of PPs may be associated with the discovery of clinically significant (likely pathogenic or pathogenic) variants in the corresponding genes, we performed an annotation of all the variants discovered in these genes in the analysed cohort. The annotation was performed using the InterVar tool, which applies the ACMG (2015 guidelines) criteria for variant classification. The analysis revealed potentially clinically significant variants, marked as Pathogenic or Likely Pathogenic in genes containing PPs (Table 1). Pathogenic criteria were triggered in InterVar due to the fact that these variants either cause frameshift or loss of splice site. All the variants presented in Table 1 lie on the intron–exon junctions, indirectly supporting the idea of their PP origin.

**Figure 2:**
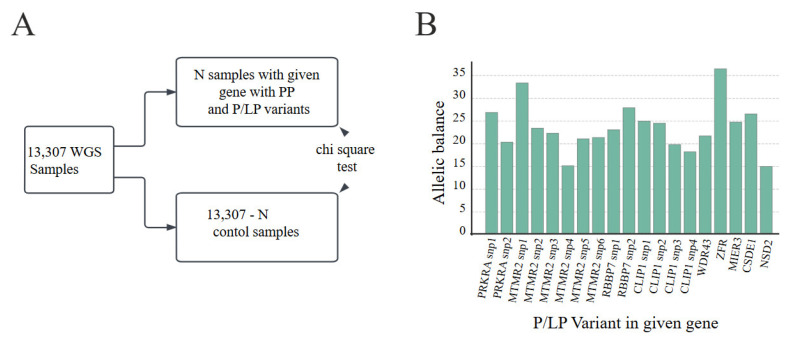
Experiment design and allele balance for clinically significant variants associated with pseudogenes. (**A**) The diagram illustrates the statistical setup to determine whether a clinically significant variant (P/LPV—pathogenic/likely pathogenic variants) belongs to a pseudogene. The 13,307 whole-genome sequencing (WGS) samples are divided into those containing a pseudogene in a particular gene and those without a pseudogene. This statistical analysis was performed for each gene in which both a clinically significant variant and a processed pseudogene were found. (**B**) The bar chart shows the allele balance for clinically significant variants in genes with PPs.

To confirm that these variants (n = 25, Table S2) were indeed present in the PPs and not in a functional gene, we split the cohort (n = 13,307) into two groups for each variant: one group consisted of samples containing the processed pseudogene, while the other group contained samples without it (the number of samples with PPs and clinically significant variants is indicated in Table 1) (Figure 2A). This split permitted a statistical evaluation of the occurrence of clinically significant variants specifically in the PPs. Indeed, potentially clinically significant variants were associated with a group of samples with identified PPs (Table S2). In reality, all potentially clinically significant variants in the analysed genes were found in samples with detected PPs. This strongly suggests that the variants are localised in the PPs and not in the parental genes. Therefore, we can state that these are false positive variants that have no real clinical significance.

In addition, we analysed the AB for each of the variants of interest that was clinically significant and was found in genes with PPs (n = 25, Table S2). In all cases, the AB was approximately 25–30% (Figure 2B), supporting the conclusion that these variants are located within the PPs. This AB is consistent with the expected pattern for variants in PPs and increases the likelihood that these variants are non-pathogenic in nature. Overall, these observations support the hypothesis that PPs harbour deleterious variants that have been incorrectly assigned to parental genes.

### 2.3. Rate of Processed Pseudogene-Associated Errors Varies Significantly Between Common Pipelines for Germline Variants Calling

**Figure 3:**
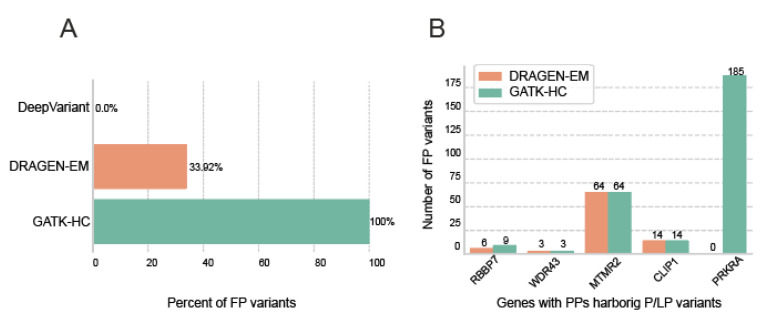
Comparison of the performance of different variant calling pipelines in samples with gene-processed pseudogene pairs. (**A**). The bar chart shows the percentage of false positive (FP) variants detected by different analysis pipelines. (**B**). The bar chart breaks down the number of likely pathogenic FP variants detected by GATK-HC or DRAGEN-EM for different genes.

In the final step, we decided to evaluate how different standard germline variant detection pipelines handle samples with problematic variants in PPs. We selected samples that contained both PPs and clinically significant variants (656 unique samples from Table 1). These samples were re-analysed using the most popular pipelines for germline SNP calling, namely, GATK-HC, DRAGEN-EM, and DeepVariant, starting with the raw FASTQ data (Figure 3).

GATK-HC detects more false positive variants than DRAGEN-EM, which indicates the higher precision of DRAGEN-EM compared to GATK-HC. In addition, DeepVariant outperforms both DRAGEN-EM and GATK-HC and has the highest precision in variant detection among the three tools. Remarkably, DeepVariant did not detect any of the false positive variants (Figure 3A).

When analysing individual genes, we found that DRAGEN-EM detected the same false positive variants as GATK-HC in genes such as RBBP7, WDR43, MTMR2, and CLIP1 (Figure 3B). However, DRAGEN-EM did not detect any variants in PRKRA, even though samples containing this gene made up a significant portion of the dataset. We analysed the bam files produced by the GATK best practice pipeline and DRAGEN-EM pipeline (Figure S3). Reads spanning PRKRA intron–exon junctions were uniquely mapped to chr6_GL000253v2_alt contig (CIGAR string 150M) by dragmap, whereas BWA-MEM, used in the best practice pipeline, created multimapper pair, which allowed for HaplotypeCaller to call INDEL at that position on chr2. Interestingly, DeepVariant correctly did not call these variants, even though the BWA-MEM bam files were used as inputs.

This comparative analysis emphasises the importance of pipeline selection in variant detection, especially in regions containing PPs. Certain pipelines can reduce the probability of false positive discoveries. In particular, DeepVariant showed the best results in the setting used by successfully excluding pseudogene variants.

## 3. Discussion

In this study, we have shown that the presence of PPs can affect the accuracy of variant identification when analysing whole-genome sequencing (WGS) data. By analysing the structural variations in 13,307 WGS samples, we successfully identified PPs and created a comprehensive database of genes with potential PPs in the cohort.

Interestingly, the majority of genes identified by the proposed algorithm (177 out of 234) were not previously annotated as PPs in the reference genome hg38. This result is to be expected, considering that the Russian population we studied has not been comprehensively analysed in previous genomic studies. Previous studies have primarily focused on other populations and have not thoroughly investigated pseudogene content in Russian cohorts [14,15]. This demonstrates the importance of including diverse populations in genomic research to improve reference genome annotations and increase the accuracy of genetic analyses. On the other hand, the algorithm identified PPs in the PRKRA, SKA3, and SMAD4 genes, which is consistent with the results of recent large-scale studies [4,16].

Although there have been analogous studies on the identification of PPs [17,18], these do not focus on the effects of these PPs on the annotation of pathogenic and likely pathogenic variants. The current study fills this gap by demonstrating how PPs can lead to false positive reports of clinically significant variants in parental genes. Overall, 4.9% of our cohort harbour P or LP variants falsely called due to pseudogene presence. The statistical analyses and AB assessments strongly support the notion that these variants originate from PPs, rather than functional gene sequences. This misclassification can lead to misinterpretation in clinical practice, which emphasises the clinical relevance of the results obtained.

Overall, the prevalence of PPs in the cohort was significant, with 73% of individuals in our cohort harbouring at least one PP, emphasising the importance of considering PPs in the genomic analyses. For example, Gnomad v4 contains 24 long indels at the position chr2:178436319, which corresponds to the one of the PRKRA intron–exon junction. The data presented in the current study suggest that these variants are most likely artefacts of PRKRA PP, which has a high frequency in our cohort (47%). Similar indel patterns could be observed at other PRKRA intron–exon junctions in the Gnomad database. Previous studies have reported frequencies of PPs in other populations [4,19], but the numbers can vary greatly depending on the population studied and the detection methods used.

Apart from introducing errors into genetic studies, PPs may have biological significance. The literature suggests that at least 10% of PPs retain their transcriptional activity because they can be randomly integrated near RNA polymerase II promoters [20]. Consequently, PPs containing clinically significant variants could affect gene expression and potentially impact phenotype. In addition, processed pseudogene insertions have been linked to disease. Thus, retrotransposition events involving PPs can disrupt normal gene function when they occur in critical genomic regions. Such insertions have been shown to inactivate tumour suppressor genes and promote cancer development [21]. These PPs may also contribute to genomic instability by causing structural variations that can lead to diseases such as chronic granulomatous disease and various cancers.

The comparative analysis of the different variant detection pipelines revealed significant differences in their ability to handle issues related to PPs. DeepVariant performed best by avoiding false positive calls in regions with PPs. This superior accuracy is consistent with other variant detection studies where DeepVariant performed better due to its machine learning-based approach. This highlights the need to carefully select analysis tools for genetic studies, especially in clinical applications where accuracy is crucial. The false positive identification of clinically significant variants can lead to incorrect diagnoses, unnecessary medical interventions, and psychological distress for patients. Given the prevalence of PPs and their potential impact on genetic analysis, it is critical to implement methods that accurately distinguish between variants in PPs and those in parental genes. Improving genome annotations, particularly in underrepresented populations, and developing more accurate analysis methods will help to reduce errors and improve the quality of genetic research and clinical interpretations.

The present study had several limitations in the process of PP identification, as well as in assessing their influence on the analysis. Firstly, despite detecting the presence of PPs, we did not address the issue of the genomic position where integration occurs. Nevertheless, integration of PPs in different genomic locations can lead to varying consequences. Secondly, we did not differentiate between different PPs of the same gene, which can arise from different isoforms. Preliminary data suggest that, for example, the PRKRA gene has several different PPs containing different exons. Also, the current study relies on automatic ACMG classification, which is one of the methods used for evaluating the pathogenicity of SNP/INDELs. Other approaches might work better with PP-originating artefacts.

This study demonstrates the importance of considering PPs when analysing genomic data and the prevalence of non-reference PPs in the population. DeepVariant performed better than the other options tested. DRAGEN-EM performed well only when a PP was present in the reference genome on the alt contig. The majority of identified false positive calls associated with PPs were at intron–exon junctions, which may prove to be a useful marker for their identification in the absence of structural variation calls.

## 4. Materials and Methods

### 4.1. Sample Collection and Whole-Genome Sequencing

To assess the possibility of detecting PPs using standard 30x WGS data, we used samples collected by “Evogen” during routine sequencing of the healthy Russian population. The quality metrics of the cohort (mean coverage, mapping rate, and percent of identified SNP/INDELS found in dbSNP) are presented in Supplementary Figure S4. The selected WGS cohort contained 13,307 individuals.

### 4.2. Library Preparation and Sequencing

Library preparation and sequencing were performed as previously described [22]. DNA extraction was performed by spin column using the Qiagen QIAamp DNA Blood Kit (Cat. No. 51106) from whole blood according to the manufacturer’s protocol. The amount of DNA was measured fluorometrically using Qubit4 (Thermo Fisher Scientific, Waltham, MA, USA)/Denovix (DeNovix Inc, Wilmington, DE, USA). Only high-quality genomic DNA (OD260/OD280 = 1.8–2.0, OD260/OD230 > 2.0) were used for subsequent library preparation. Library preparation was performed with a PCR-free enzyme fragmentation protocol (MGIEasy FS PCR-Free DNA Library Prep Set, Cat. No. 1000013455) using 800–1200 ng gDNA. The size distribution of the inserts was 400–600 bp. WGS library preparation was performed both manually and automatically. Whole-genome sequencing was performed using DNBSEQ-G400 (MGI Tech Co., Ltd., Shenzhen, China) with FCL PE150 (Cat. No. 1000012555), FCL PE200 (Cat. No. 1000013858), and DNBSEQ-T7, according to the manufacturer’s protocol. The average sequencing depth was 30x.

### 4.3. Variant Calling

The initial analysis of the samples was performed using a modified version of the GATK Best Practices workflow. The modification included a change from BWA-MEM to minimap2 (v2.17-r941) and a change from GATK HaplotypeCaller to GATK HaplotypeCaller spark (v4.6.0.0). For the evaluation of the results, publicly available pipelines were used for the reanalysis of selected samples: GATK Best Practices (https://github.com/broadinstitute/warp/tree/develop/pipelines/broad/dna_seq/germline/single_sample/wgs, accessed on 1 May 2024), GATK Dragen Equivalence Mode (DRAGEN-EM), and BWA-MEM with DeepVariant (v1.5.0). In short, the Broad Institute’s standard GATK pipeline includes BWA-MEM (0.7.15-r1140) as a read aligner, Picard tools for duplicate labelling, BQSR for base quality score recalibration, and HaplotypeCaller as a variant caller; all tools belong to the GATK:4.6.0.0 toolkit. The Broad Institute also offers a GATK DRAGEN-EM pipeline, which we used as a second option. This pipeline consisted of DRAGMAP (an open-source DRAGEN mapper, v1.2.1), the same Picard tools for duplicate marking, BQSR, and HaplotypeCaller. As a third option, we used BAM files after the BWA-MEM alignment step and performed variant calling with DeepVariant. For all pipelines, the recommended hg38 reference was used (https://console.cloud.google.com/storage/browser/gcp-public-data--broad-references/hg38/v0/, accessed on 1 May 2024).

### 4.4. Structural Variation Detection

Structural variations (SVs) were detected using Smoove (v0.2.6) [23], an optimised wrapper for the LUMPY SV caller (v0.3.1) [24] applied to the Minimap2-aligned data. Smoove efficiently identifies different types of SVs, including deletions, duplications, insertions, inversions, and translocations, by integrating information about read pairs, split read, and coverage depth.

### 4.5. Processed Pseudogene Detection Pipeline

We developed a pipeline for the detection of PPs based on the structural variant data obtained from Smoove. The pipeline searches the VCF files generated by Smoove for multiple intronic deletions within each gene. By identifying genes with intronic deletions, the pipeline determines candidate genes with PPs (Figure S1). The code for Smoove vcf file processing is available at (https://gitlab.com/Artemlin6231/pseudogene_detecting, accessed on 1 May 2024).

To validate our method for detecting PPs, we compared the identified PPs with those annotated in the GENCODE (v46) database (https://ftp.ebi.ac.uk/pub/databases/gencode/Gencode_human/release_46/gencode.v46.chr_patch_hapl_scaff.annotation.gtf.gz, accessed on 1 May 2024) and reported in the recent literature. We assessed the accuracy by calculating the allele balance and analysing the ratio of read depth per gene between exons and introns.

### 4.6. Identification of Likely Pathogenic Variants Associated with Processed Pseudogenes

Variants identified within genes with PPs in the same samples were annotated according to the 2015 ACMG guidelines [6] using InterVar software (v2.2.1) [7], according to the developer’s manual. Variants were normalised prior to annotation with open cravat [25].

### 4.7. Quantitative Analysis of Pseudogene-Associated Errors in Variant Calling

To quantify the potentially clinically relevant errors caused by PPs in variant calling, we re-analysed the reads mapped to genes with PPs and potentially clinically significant SNPs using alternative germline calling pipelines based on DRAGEN-EM and DeepVariant callers. By comparing the results of GATK-HC, DRAGEN-EM, and DeepVariant, we were able to determine the extent to which each pipeline was affected by pseudogene interference.

### 4.8. Statistical Analysis

To assess the statistical significance of clinically significant variants belonging to PPs, we used the comparative statistical chi-squared test (χ²). Significance was determined at a threshold of *p* < 0.05.

## Figures and Tables

**Table 1:** Number of samples with clinically significant variants, according to ACMG criteria, detected in samples with genes containing PPs.

Gene	Samples with LPV	Samples with PV	Unique Positions	Total Samples with PPs
*PRKRA*	438	23	2	6234
*MTMR2*	181	0	4	275
*CLIP1*	42	0	4	61
*WDR43*	23	0	2	158
*RBBP7*	13	2	2	18
*SMAD4*	2	0	2	152
*MATR3*	1	0	1	497
*SF1*	1	0	1	1
*NDFIP1*	1	0	1	1
*DLG3*	1	0	1	1
*CSDE1*	1	0	1	1
*ZFR*	1	0	1	1
*MIER3*	1	0	1	1
*NSD2*	1	0	1	1

## Data Availability

Restrictions apply to the availability of these data. Data were obtained from Evogen LLC and are available from the authors with the permission of Evogen LLC. List of discovered processed pseudogenes with corresponding frequencies is included in the Supplementary Materials.

## Supplementary Materials

The following supporting information can be downloaded at https://www.mdpi.com/article/10.3390/ijms26010363/s1.
