# Supplementary figures and images for: Quantitative Analysis of Pseudogene-Associated Errors During Germline Variant Calling
Source: Int J Mol Sci. 2025 Jan 3;26(1):363. doi: 10.3390/ijms26010363 (PMC11719938; doi:10.3390/ijms26010363)

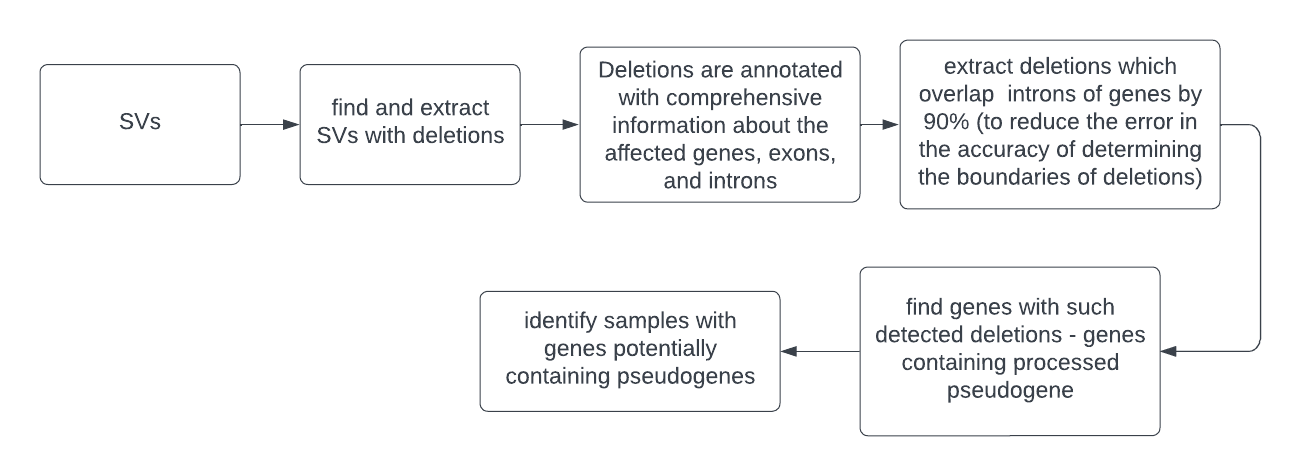

Supplement: Supplementary file 1 [file ijms-26-00363-s001.zip › Supplementary Figure S1.png]

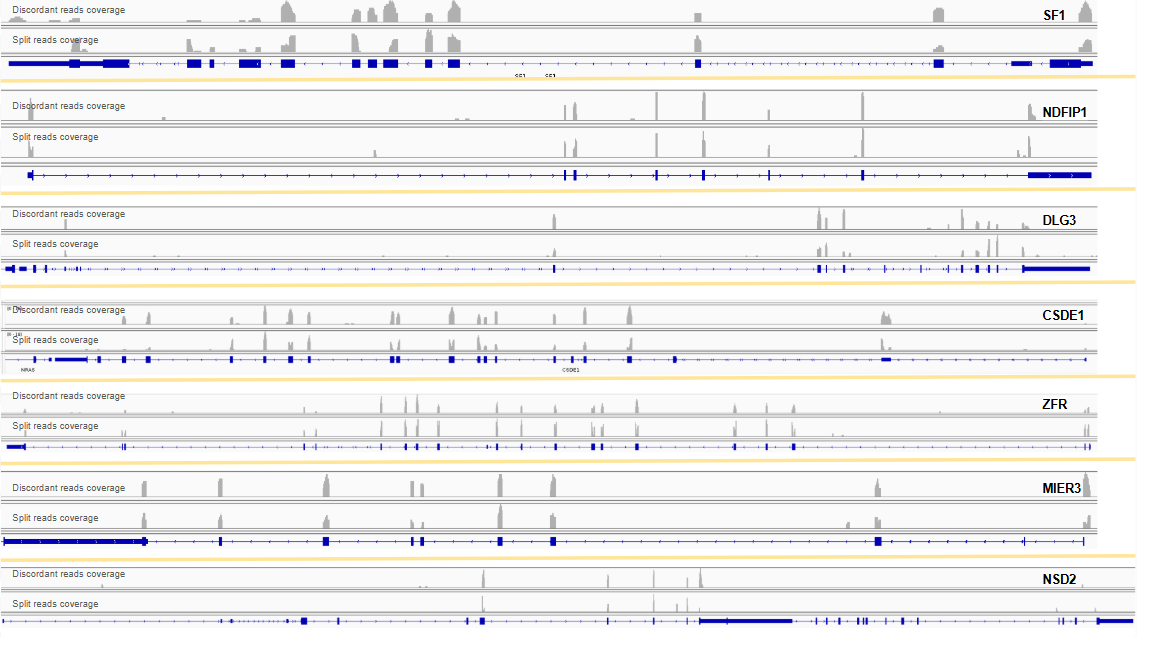

Supplement: Supplementary file 1 [file ijms-26-00363-s001.zip › Supplementary Figure S2.png]

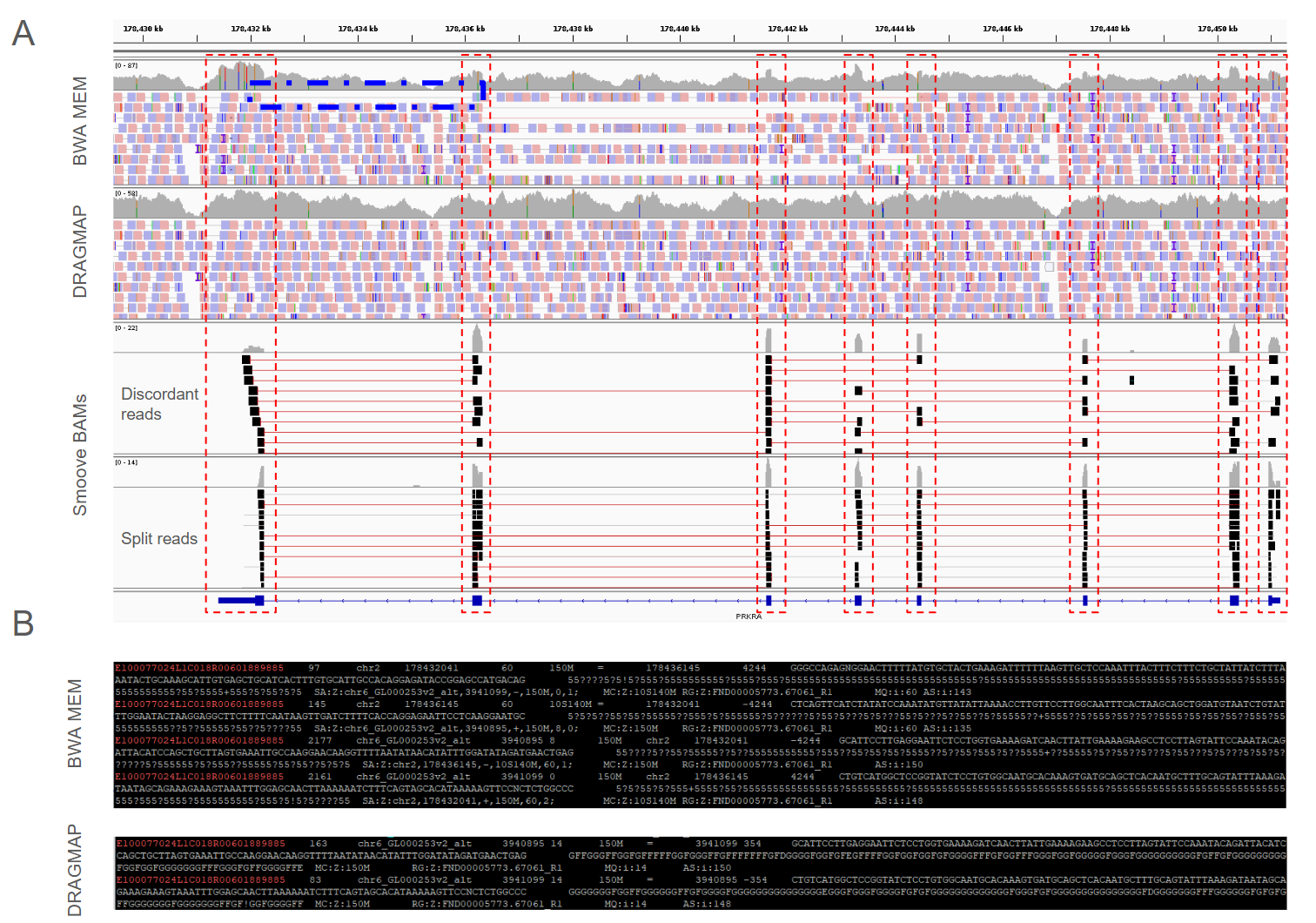

Supplement: Supplementary file 1 [file ijms-26-00363-s001.zip › Supplementary Figure S3.png]

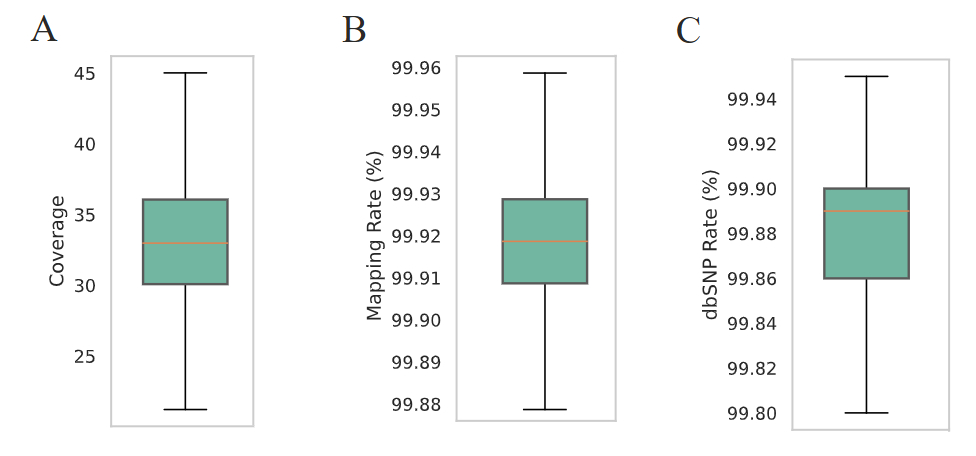

Supplement: Supplementary file 1 [file ijms-26-00363-s001.zip › Supplementary Figure S4.png]
